# Linking functional response and bioenergetics to estimate juvenile salmon growth in a reservoir food web

**DOI:** 10.1371/journal.pone.0185933

**Published:** 2017-10-11

**Authors:** Craig A. Haskell, David A. Beauchamp, Stephen M. Bollens

**Affiliations:** 1 U.S. Geological Survey, Western Fisheries Research Center, Cook, Washington, United States of America; 2 U.S. Geological Survey, Western Fisheries Research Center, Seattle, Washington, United States of America; 3 Washington State University, School of the Environment and School of Biological Sciences, Vancouver, Washington, United States of America; North Carolina State University, UNITED STATES

## Abstract

Juvenile salmon (*Oncorhynchus* spp.) use of reservoir food webs is understudied. We examined the feeding behavior of subyearling Chinook salmon (*O*. *tshawytscha*) and its relation to growth by estimating the functional response of juvenile salmon to changes in the density of *Daphnia*, an important component of reservoir food webs. We then estimated salmon growth across a broad range of water temperatures and daily rations of two primary prey, *Daphnia* and juvenile American shad (*Alosa sapidissima*) using a bioenergetics model. Laboratory feeding experiments yielded a Type-II functional response curve: *C* = 29.858 *P* *(4.271 + *P*)^-1^ indicating that salmon consumption (*C*) of *Daphnia* was not affected until *Daphnia* densities (*P*) were < 30 · L^-1^. Past field studies documented *Daphnia* densities in lower Columbia River reservoirs of < 3 · L^-1^ in July but as high as 40 · L^-1^ in August. Bioenergetics modeling indicated that subyearlings could not achieve positive growth above 22°C regardless of prey type or consumption rate. When feeding on *Daphnia*, subyearlings could not achieve positive growth above 20°C (water temperatures they commonly encounter in the lower Columbia River during summer). At 16–18°C, subyearlings had to consume about 27,000 *Daphnia* · day^-1^ to achieve positive growth. However, when feeding on juvenile American shad, subyearlings had to consume 20 shad · day^-1^ at 16–18°C, or at least 25 shad · day^-1^ at 20°C to achieve positive growth. Using empirical consumption rates and water temperatures from summer 2013, subyearlings exhibited negative growth during July (-0.23 to -0.29 g · d^-1^) and August (-0.05 to -0.07 g · d^-1^). By switching prey from *Daphnia* to juvenile shad which have a higher energy density, subyearlings can partially compensate for the effects of higher water temperatures they experience in the lower Columbia River during summer. However, achieving positive growth as piscivores requires subyearlings to feed at higher consumption rates than they exhibited empirically. While our results indicate compromised growth in reservoir habitats, the long-term repercussions to salmon populations in the Columbia River Basin are unknown.

## Introduction

Freshwater food webs are important for juvenile salmon (*Oncorhynchus* spp.) as they migrate seaward. In the Columbia River Basin (CRB), main-stem food webs have been drastically altered from river impoundment, the introduction of non-native species, and supplementation with hatchery conspecifics [1]. The effects of these food web changes are mostly unstudied but are likely important for juvenile salmon growth and their ultimate survival. Juvenile salmon survival is directly influenced by growth, with larger individuals less susceptible to predation at critical early life history stages [2]. Greater size achieved during early life stages can confer a survival advantage through the lifespan of a population [3,4]. The growth of juvenile salmon during this time is a function of water temperature, consumption, and prey quality [5,6]. Therefore, examination of these factors and their ability to constrain life-stage specific growth is warranted.

The rearing capacity of lower Columbia River food webs for juvenile salmon remains largely unstudied. The CRB has more than 200 dams, many of which have elaborate collection and bypass systems to aid juvenile salmon downstream migration [7], and researchers have focused mostly on downstream salmon passage at main-stem dams and its relation to survival rather than food web interactions. If indeed main-stem food webs are important for juvenile salmon, they are probably particularly important for fall Chinook salmon (*O*. *tshawytscha*) juveniles that exhibit an extended residence in main-stem habitats relative to other juvenile salmon life histories [8]. For example, juvenile spring Chinook salmon rear in tributary habitats and emigrate as yearlings on the ascending limb of the hydrograph. Juvenile fall Chinook salmon, also known as subyearlings, are more reliant on main-stem habitats and food webs because they migrate more slowly, at a smaller size, and at lower flows relative to other juvenile salmon [9]. Therefore, reservoir food webs in the lower CRB, while utilized by all juvenile salmon to some degree, are perhaps most important to subyearlings.

Run-of-the-river reservoirs in the lower Columbia River exhibit a high degree of spatiotemporal variability in the potential prey base for subyearlings. The prey base fluctuates on an annual and seasonal basis [10,11], but also longitudinally from upstream (tailrace) to downstream (forebay) [12]. Therefore, it is challenging to generalize the diet of subyearlings and other planktivores in the lower Columbia River due to the prevalence of run-of-the-river-reservoirs and the prey base they support. Thus, although examination of the rearing capacity of food webs for juvenile salmon is warranted, models need to be spatially and temporally explicit.

There is concern that reservoir food webs in the Columbia River are unable to meet the energetic demands of subyearlings given the large numbers of hatchery salmon and non-native juvenile American shad (*Alosa sapidissima*) that also rear there [1]. During summer, subyearlings in the lower Columbia River primarily consume *Daphnia* [12–14], but *Daphnia* decline dramatically in early August which is about the time young shad begin feeding. In 2015, nearly 140 million hatchery reared juvenile salmon were released into the CRB, but there are many more juvenile shad. In John Day Reservoir alone, as many as 112 million juvenile shad are produced annually, with many more originating in other Snake and Columbia River reservoirs and the Columbia River Estuary [15]. Juvenile shad consume up to 80% of the available zooplankton production in John Day Reservoir during the time that subyearlings are also migrating seaward [16]. It is unknown if reductions in *Daphnia* forage influence the consumption rate of subyearlings.

Functional response models can provide a fundamental framework for studying predator-prey interactions within food webs. The functional response model describes predator consumption as a function of prey density. Three primary functional response models have been identified. A Type-I response exhibits a linear increase in consumption with an increase in prey density. Type-I responses are characteristic of filter feeders and a spatially homogenous prey base. A Type—II response increases linearly initially but reaches an asymptote as handling time begins to limit predator consumption. Type-II responses are indicative of planktivores that forage in patchy or prey limited environments [17]. A Type-III or sigmoid response is exhibited by organisms that switch between prey types. There are no functional response models for juvenile Chinook salmon.

Although fine-scale growth is difficult to measure empirically given the transient nature of juvenile salmon in large rivers, it can be estimated using bioenergetics if consumption is known. The use of bioenergetics models in fisheries has increased due to the capacity of these models to address complex questions while reducing the expense and complications of field sampling [18]. Bioenergetics models are energy balance equations that can be used to estimate the growth or consumption of a fish when one of these parameters is known or measured empirically. Although the most common use of bioenergetics models is to estimate consumption using empirical growth estimates, the model can be rearranged to estimate growth using empirically derived consumption estimates [19]. Consumption and associated evacuation rates can be estimated empirically by sampling fish stomach contents at regular intervals over a 24-h period [20]. These empirical consumption estimates can then be used as inputs to estimate fish growth using bioenergetics models. Empirical consumption estimates for subyearlings migrating through lower Columbia River reservoirs during July and August have been recently estimated and provide an opportunity to relate consumption to growth [14]. These estimates generally indicated high consumption rates, but the relation between consumption, growth, and the high water temperatures at which subyearlings emigrate has not been evaluated.

Water temperature is also an important determinant of juvenile salmon growth [21,22]. Increased water temperatures at the lower end of a species thermal range increase growth, however increased temperatures at the upper end of the thermal range can decrease growth. Subyearlings can exhibit decreased growth at water temperatures exceeding 21°C [23]. However, increases in prey consumption or prey energy density can counteract the negative energetic effects of higher water temperatures to some extent [24,25]. Subyearlings in the lower CRB migrate seaward through reservoirs when water temperatures are approaching their critical thermal maxima (20.9°C; [26]). During summer, subyearlings in the lower CRB switch from consuming *Daphnia* to juvenile American shad (which have a higher energy density) after *Daphnia* diminish [14]. Here, we investigate if subyearlings can compensate for the deleterious effects of high water temperature on growth by switching from planktivory to piscivory during summer.

We estimated the consumption and growth of subyearlings as they migrate through the lower Columbia River during July and August—a time when they are subject to feeding competition from juvenile American shad, a dynamic prey base, and water temperatures approaching the upper end of their thermal tolerance. We focused our laboratory experiments on the seasonal period (July and August) when subyearlings are preying on *Daphnia* in the lower Columbia River. We first conducted laboratory functional response trials to estimate the feeding response of subyearling Chinook salmon to changes in *Daphnia* density. Next, we used empirical *Daphnia* densities from previous field studies to predict subyearling consumption using our functional response curve. Finally, we used a bioenergetics model to estimate growth changes associated with increasing water temperatures and a switch from planktivory to piscivory.

## Materials and methods

### Study area

**Fig 1:**
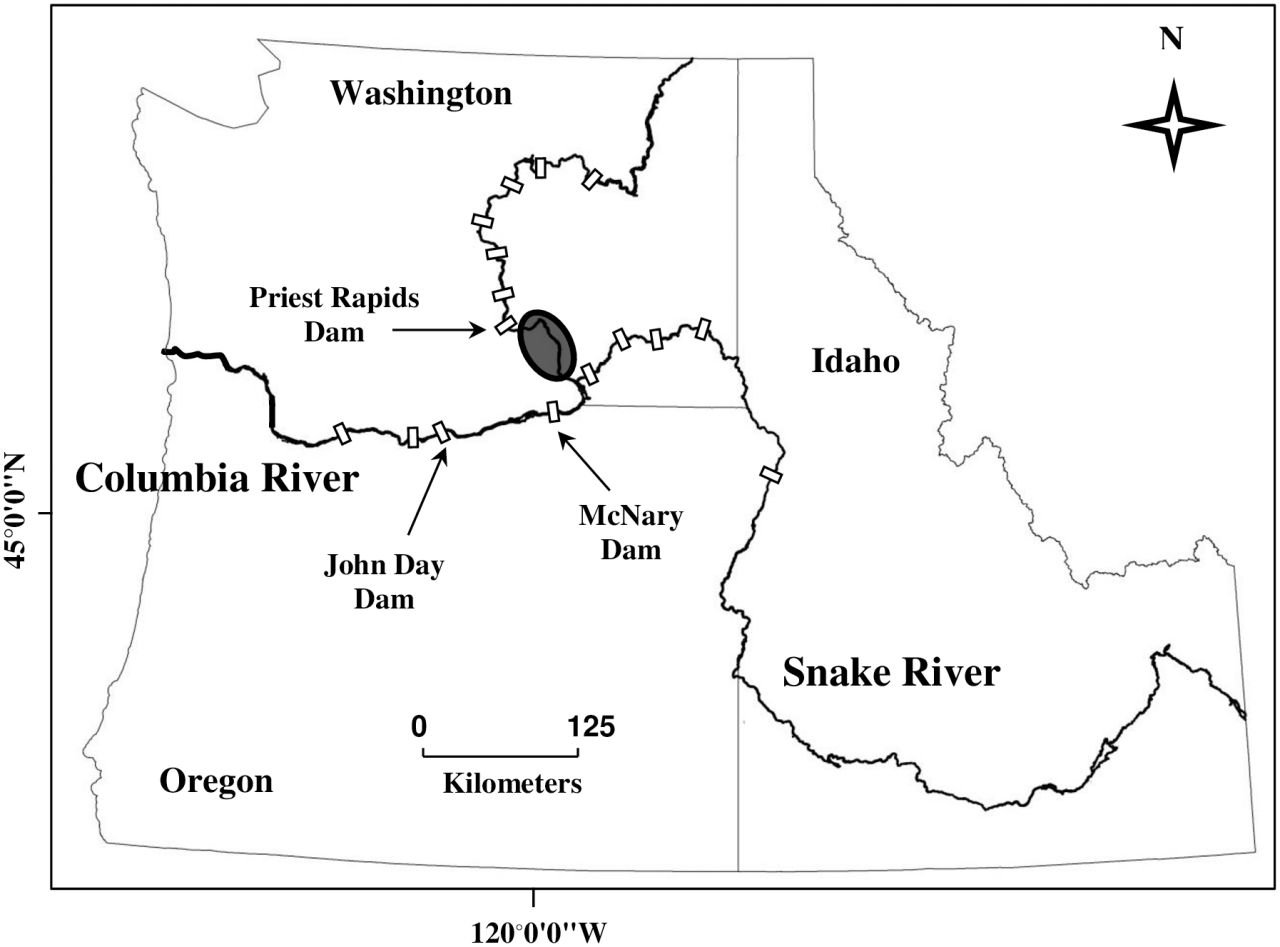
Map of the Columbia River basin depicting mainstem hydroelectric dams (white rectangles) and the Hanford Reach (shaded oval), an important spawning area for fall Chinook salmon.

Empirical data for this study were collected from John Day and McNary reservoirs, the third and fourth upstream reservoirs on the lower Columbia River. Together, John Day (river kilometer (rkm) 348) and McNary (rkm 471) dams impound a 221-km stretch of the lower Columbia River from Rufus, Oregon upstream past the Snake and Columbia River confluence to Pasco, Washington (Fig 1). Upstream of Pasco, the Columbia River is unimpounded for 70 km to Priest Rapids Dam. This stretch of river, commonly referred to as the Hanford Reach, is home to the largest naturally reproducing stock of fall Chinook salmon in the Columbia Basin [27]. Therefore, McNary and John Day reservoirs are important downstream rearing areas for both Hanford Reach fall Chinook juveniles and Endangered Species Act (ESA)-listed fall Chinook originating in the Snake River [28]. We chose John Day and McNary reservoirs because of their importance for juvenile salmon rearing, and because a relatively large amount of empirical data on *Daphnia* densities, water temperature, and subyearling consumption exist from past studies.

### Ethics statement

The Western Fisheries Research Center is exempt from IACUC approval because fish are exempt from the U.S. Department of Agriculture definition of animals under the Animal Welfare Act. Because our laboratory specifically receives no funds from PHS agencies (The National Institute of Health, Centers for Disease Control and Prevention, and the Food and Drug Administration), we are not required to obtain IACUC approval. Fish for this experiment were obtained from the Willard National Fish Hatchery operated by U.S. Fish and Wildlife Service. The U.S. Geological Survey is required to obtain state permits for the transport of fish to our facility for experimentation. State of Washington permits require that we dispose (euthanize) fish after the conclusion of experiments, however, protocols for euthanizing fish are not stipulated. Although not required by state permitting, we euthanized fish by administering a lethal dose of MS-222.

### Functional response laboratory trials

To examine the relationship between subyearling Chinook salmon consumption and *Daphnia* density, we used 61 cm (24 inch) cylindrical trial tanks ranging from 53.1 to 61.0 L. Five hundred ‘Tule’ strain juvenile fall Chinook were obtained from the Little White Salmon fish hatchery in Willard, Washington on April 10, 2014. A random sample of forty fish had a mean fork length (FL) of 71.7 mm (range = 63–86 mm) and a mean weight of 3.8 g (range = 2.4–7.2 g). Before trials, salmon were held in large cylindrical tanks supplied with aerated 7°C well water and fed 5% of their body weight per day with BioClark’s Fry 1.5 mm feed. Trials were run at temperatures similar to those experienced by subyearlings in John Day Reservoir during field consumption trials conducted by Haskell et al. ([14]; 20°C). About 10 days before running trials, single juvenile salmon were moved to trial tanks and brought up to trial temperature at a rate of 1–2°C a day and fed frozen zooplankton collected from Lower Granite Reservoir, Washington for 3 days to acclimate them to the prey used in trials. Fish were starved for the final 48 h leading up to the trials.

Prey for the trials were live *Daphnia pulex*. *Daphnia* were obtained from Sachs Systems Aquaculture and then cultured in 21 L aquaria with Biofilm filters. Water temperatures in the aquaria were ambient with air temperature held constant at 21.1°C (70°F). Photoperiod mimicked conditions in John Day Reservoir during mid-August, when daylight was from 0600 to 2010 hr. We fed *Daphnia* an equal-parts mixture of dried *Chlorella*, *Spirulina* algae, and activated dry yeast. To each aquarium, 0.0625 g of the mixture was added when increasing water clarity generally indicated the need for more feed. *Daphnia* were seeded to other aquaria as required to increase densities. We used Seminole Rams-Horn snails (*Planorbella duryi*) to help reduce metabolites and subsequent fouling of aquarium water. Snails were periodically culled to maintain about 20 per tank. *Daphnia* were culled during water changes to keep cultures from overpopulating and crashing.

Individual functional response trials were run for 10 min, beginning from the time of first *Daphnia* capture [29,30] as monitored by an overhead video camera connected to a monitor (Speco technologies model CVC-320WP). Light intensity within trial tanks was constant, but ranged from 11.8 to 24.8 lx across tanks. We introduced *Daphnia* to achieve densities ranging from 0.1 to 161.4 · L^-1^. We wanted to populate the functional response curve with an ecologically relevant range of *Daphnia* densities from John Day Reservoir [10] but also some higher densities to determine at what densities the curve would ‘flatten out’. We defined the ecologically relevant range of *Daphnia* densities as those < 40 · L^-1^. For low-density trials (< 5 · L^-1^), individual live *Daphnia* were counted at the beginning of the trial. For higher density trials, we used the number of uneaten *Daphnia* filtered from the trial tank and preserved in ethanol at the end of the trial to determine trial densities. Initial trials were run at densities based on the availability of cultured *Daphnia*. After initial trials, we ran trials at higher or lower density to cover the range of densities reported for John Day Reservoir when subyearlings are present. *Daphnia* were filtered through a 500 micron mesh net to remove smaller *Daphnia* that were not in the size range consumed by subyearling Chinook salmon [12].

During trials, prey density was maintained by adding 10, 20, or 30 *Daphnia* via a feeder hose based on the real-time observations of consumption rate using the camera system. The feeder hose was connected to a standard funnel located behind an office partition to minimize shadows that could affect salmon behavior during the trial. At the conclusion of a trial, fish were immediately euthanized in MS-222 and frozen. Fish guts were later examined and the number of *Daphnia* in the stomach was counted. Consumption rate was determined by counting the number of *Daphnia* in individual stomachs and dividing by the trial time. At the conclusion of a trial, tank water was filtered through a 63 micron mesh to collect uneaten *Daphnia*. In cases where the total number of uneaten *Daphnia* was large (> 1,300), the sample was diluted to a standard dilution and five 1-ml aliquots of at least 50 individuals were counted. The mean of the aliquots was used to estimate the total number of uneaten *Daphnia* [31].

We compared linear and non-linear regression models to evaluate whether our data indicated a Type-I or Type-II functional response, respectively, and then used the appropriate model as a predictor of consumption rate based on prey density. Linear regression models were developed using PROC REG and nonlinear regression models were developed using PROC NLIN (SAS 2002).

The linear equation form was:

C=aP (1)

where *C* = the consumption rate of the predator, *P* = *Daphnia* density, and *a* is the slope of the consumption rate.

The nonlinear equation form was:

$$C=\beta_{0}P\;{\left(\beta_{1}+P\right)}^{-1}$$ (2)

where *β*_0_ is the maximum consumption rate and *β*_1_ is the prey density at which consumption reaches half of its maximum [32]. We used our resulting functional response curve to predict subyearling consumption from empirical *Daphnia* densities.

### Functional response model evaluation

We developed two models from the laboratory data. The first model we developed from the entire range of *Daphnia* densities and subyearling consumption rates that we measured. The second model we developed from the ecologically relevant range of *Daphnia* densities (< 40 · L^-1^) in McNary and John Day reservoirs as determined from the literature. We compared our non-linear (Type-II functional response) and linear (Type-I functional response) models using corrected Akaike’s Information Criteria (*AIC*_*c*;_ [33]). Here,

AICc=N*ln(RSSN)+2K+2K(K+1)N−K−1 (3)

where *K* = the number of parameters +1, *N* = the number of trials, and *RSS* = the error sum of squares. The more appropriate model was selected based on the lower *AIC*_*c*_ score with *AIC*_*c*_ differences > 10 indicating little support for model similarity. Models were also selected by how they performed within the range of ecologically realistic *Daphnia* densities from the literature. We used Cook’s *D* statistic to examine the influence of individual points on the original linear model. We did not test a Type-III response based on the general appearance of the data.

### Empirical *Daphnia* densities

To determine empirical *Daphnia* densities in John Day Reservoir, we consulted previous studies. In June-August of 1980–1982, Rondorf et al. [12] collected zooplankton using a horizontally towed paired Miller sampler with 153 μm-mesh in McNary Reservoir. Using the same sampling gear in 1994–1996, Haskell et al. [10] collected weekly zooplankton samples in McNary Reservoir during June and July and every other week in John Day Reservoir during August–November. This collection was intended to coincide with the migration of subyearling Chinook salmon and juvenile American shad. Gilbreath et al. [34] collected monthly zooplankton in John Day Reservoir from April 1994 to September 1995 using vertical plankton tows with a Wisconsin style 80 μm-mesh net. The net was towed from either 5m to the surface (upper reservoir) or from 15 m to the surface (mid and lower reservoir). Lastly, Emerson et al. [11] collected zooplankton monthly in the forebay of John Day Reservoir from July 2009- June 2011 using a 0.5 m, 73 μm-mesh net towed vertically from 0.5 m off the bottom to the surface. We used all of these data to better understand the range of *Daphnia* densities in lower Columbia River reservoirs at the time subyearling Chinook salmon are migrating and feeding.

We estimated stomach capacity using empirical *Daphnia* densities from various sources in McNary and John Day reservoirs, predicted consumption from our functional response curve, empirical *Daphnia* weights estimated from length to weight relations [16,35], and a relation between stomach capacity in grams and fish mass derived for sockeye salmon ranging from 2–350 g [36]:

$$CP=\frac{M*\left(14.1-4.95*\text{log}(M\right)}{100}$$ (4)

Here, *CP* = capacity (g) and *M* = subyearling Chinook salmon mass (g). We estimated time to satiation (h) using empirical *Daphnia* densities from various field studies, predicted consumption rates from our functional response curve, and evacuation rates from previously conducted consumption field trials [14]. Evacuation rates for subyearlings ranged from 0.58 · h^−1^ at 20.78°C in July to 0.51 h^−1^ at 22.02°C in August. At ambient temps of 20–22°C, the empirical estimates of evacuation rates were 0.51–0.58 · h^-1^.

### Subyearling Chinook salmon bioenergetics model runs

Bioenergetics models are energy balance models which can be used to estimate the required consumption to achieve a certain growth rate [5]. The model can also be rearranged to estimate growth given observed consumption over a specific time interval where:

Growth=consumption−waste−metabolism (5)

Species-specific physiological parameters exist and are packaged in the ‘Wisconsin model’ but often rely on parameters that are borrowed from closely related species given the difficulty in deriving species-specific parameters in the laboratory [5]. The Chinook salmon bioenergetics model was originally developed by Stewart and Ibarra [37] using consumption parameters borrowed from a coho salmon (*O*. *kisutch*) model with a temperature dependent *C*_*max*_ curve peaking around 16.5°C. We incorporated newly developed temperature dependent consumption parameters specifically for subyearling Chinook salmon into the existing bioenergetics model (Table 1). These parameters suggest a temperature-dependent *C*_*max*_ curve about 4°C higher than the previous model [26]. We used % *C*_*max*_ and growth efficiency (*GE*) as relative measures of feeding performance. *C*_*max*_ is the maximum possible consumption for a fish of a given weight at temperatures where consumption is maximized. For subyearlings:

$$C_{max}=0.303*W^{-0.275}$$ (6)

where *W* = weight (g).

$$\%\;C_{max}=C/C_{max}$$ (7)

where *C* = specific consumption (g · g · d^-1^). Growth efficiency (*GE*) is defined as:

GE=(GC)*100 (8)

where *G* = specific growth (g · g · d^-1^) from the bioenergetics output.

**Table 1:** The Wisconsin bioenergetics model [5] with species specific parameters developed by Stewart and Ibarra [37] and modified consumption parameters from Plumb and Moffitt [26] used to model juvenile Chinook salmon growth.

Parameter	Juvenile Chinook salmon value	Source
**Consumption Equation**		
CA	0.303	Stewart and Ibarra (1991)
CB	-0.275	Stewart and Ibarra (1991)
CQ	5	Stewart and Ibarra (1991)
CTO	15	Stewart and Ibarra (1991)
CTM	20.93	Plumb and Moffit (2015)
CTL	24.05	Plumb and Moffit (2015)
CK1	0.36	Stewart and Ibarra (1991)
CK4	0.53	Plumb and Moffit (2015)
**Respiration Equation**		
RA	0.00264	Stewart and Ibarra (1991)
RB	-0.217	Stewart and Ibarra (1991)
RQ	0.06818	Stewart and Ibarra (1991)
RTO	0.0234	Stewart and Ibarra (1991)
RTM	0	Stewart and Ibarra (1991)
RTL	25	Stewart and Ibarra (1991)
RK1	1	Stewart and Ibarra (1991)
RK4	0.13	Stewart and Ibarra (1991)
ACT	9.7	Stewart and Ibarra (1991)
BACT	0.0405	Stewart and Ibarra (1991)
SDA	0.172	Stewart and Ibarra (1991)
**Egestion/Excretion Equation**		
FA	0.212	Stewart and Ibarra (1991)
FB	-0.222	Stewart and Ibarra (1991)
FG	0.631	Stewart and Ibarra (1991)
UA	0.0314	Stewart and Ibarra (1991)
UB	0.58	Stewart and Ibarra (1991)
UG	-0.299	Stewart and Ibarra (1991)

We used the bioenergetics model to run two sets of growth simulations for subyearling Chinook salmon. The first set was two 4-day simulations using empirical prey percentages, water temperatures, and empirical consumption rates estimated from 24-h field trials conducted on July 21–24 and August 11–14, 2013 as described in [14]. In this study, subyearlings predominately consumed *Daphnia* in July and juvenile American shad in August. Other studies have also noted the importance of *Daphnia* to juvenile salmon in the lower Columbia River during this time [12,38].

For the second set of simulations, we wanted to more broadly understand the effect of varying prey consumption, prey type, and water temperature on subyearling growth beyond the two 4-day scenarios that we originally modeled. Therefore, we ran multiple 20-d scenarios, varying the consumption rates on the two primary prey items of subyearlings, *Daphnia* and juvenile American shad, and by varying water temperature within the range experienced by subyearlings migrating through John Day Reservoir during June–August [39]. For these model runs, we used a starting weight of 20 g for subyearlings and assumed 100% by weight of the diet consisted of *Daphnia* in our planktivory simulations and 100% juvenile American shad in our piscivory simulations. In the planktivory simulations, we varied water temperature from 16–24°C in 2°C increments and varied *Daphnia* consumption from 2,000 to 32,000 *Daphnia* · day^-1^ in 5,000 *Daphnia* · day^-1^- increments. For the piscivory simulations, we used the same temperature ranges and 20-d durations, but varied consumption from 10 to 40 shad · day^-1^ at 5 shad · day^-1^ -increments. Thirty five scenarios (5 temperatures * 7 *Daphnia* consumption rates) were run for the planktivory simulations and 35 scenarios (5 temperatures * 7 shad consumption rates) were run for the piscivory simulations. For individual scenarios, water temperature and consumption rate were held constant over the 20-d model runs. Prey energy densities were collected from various sources (Table 2), and we assumed 10% of prey were indigestible [40]. Predator energy density was estimated based on a 78.8% moisture content converted to a species specific energy density for juvenile Chinook salmon of 4,730 J · g^-1^ [41]. We used a wet weight of 0.083 g for juvenile American shad prey (N = 25, mean FL = 24.4 mm, range FL = 11–40 mm) [14]. We used the midpoint of the energy density range reported for larval fish by Hanson et al. (1997) for shad energy density inputs although some of those fish had probably transitioned to the juvenile stage.

**Table 2:** Energy densities and proportions of prey items collected from juvenile fall Chinook salmon in John Day Reservoir, Columbia River from 21 July and 11 August 2013 [14]. Twelve juvenile Chinook stomachs were examined from each date.

Prey taxa	Energy Density (J/g wet)	21 July	11 Aug
*Daphnia*[42]	1,620	0.9796	0.0208
Juvenile American shad[5]	3,698	0.0001	0.7292
Other inverts[43]	4,532	0.0203	0.2500

The estimation of ‘average’ *Daphnia* wet weight was an important consideration for the estimation of subyearling growth, % *C*_*max*_, and stomach capacity (g) calculations. To estimate the wet weight of ambient (in the river) *Daphnia*, we first used a mean length from empirical estimates converted to a species-specific dry weight following Culver et al. [35]. We assumed that all *Daphnia*, both consumed and ambient, were *D*. *retrocurva*- the primary *Daphnia* species in the lower Columbia River during July and August [10,11]. We then assumed that the dry weight of *Daphnia* was 10% of wet weight [5]. However, kokanee salmon and other planktivores have a demonstrated ability to reduce the water content of *Daphnia* by about 50% after consuming them, thereby increasing the number of *Daphnia* they can consume [44]. Therefore, we assumed ambient *Daphnia* dry weight was 7% of consumed wet weight and consumed *Daphnia* had an energy density of 1,620 J/g [42].

During sampling in the early 1980’s, Rondorf et al. [12] reported a mean length for ambient *Daphnia* of 1.05 mm in July and 1.14 mm in August in contrast to Haskell et al. [10] who found a mean length for ambient *Daphnia* ranging from of 0.84 mm in the mid-1990’s during July and August. Differences in mean *Daphnia* length between the two sampling periods could be due to increased planktivory by juvenile American shad and the mysid, *Neomysis mercedis* [45]. *Neomysis* was not present during sampling in the early 1980’s and shad numbers were much lower than they are today. Nevertheless, Rondorf et al. [12] reported a 0.36 mm difference between the size of ambient *Daphnia* and those consumed by subyearlings. Therefore, we assumed a similar difference between the mean *Daphnia* length reported by Haskell et al. [10], but compensated for size selection by assuming subyearlings were consuming *Daphnia* that were 1.2 mm on average.

## Results

### Functional response model selection

**Fig 2:**
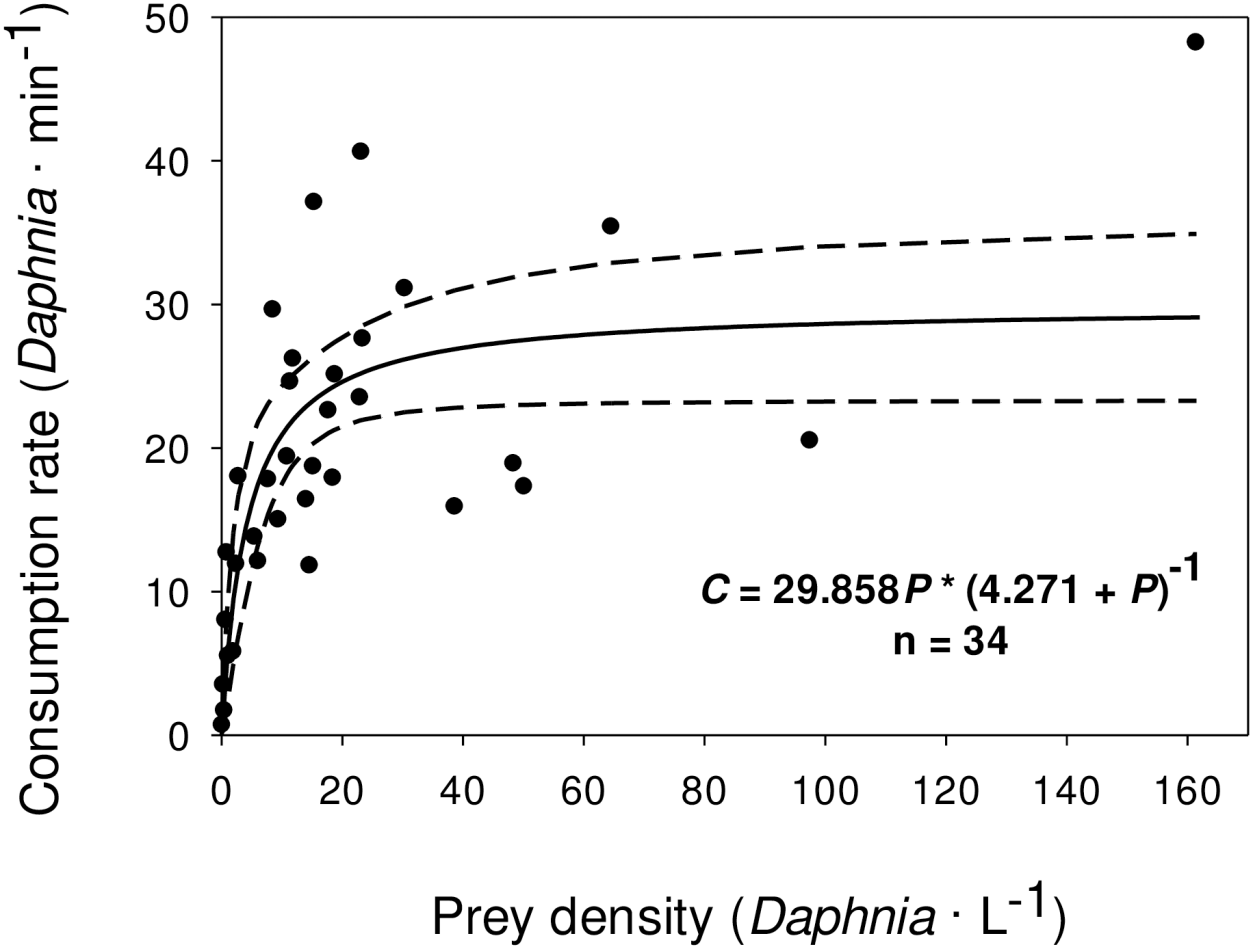
Type-II (solid line) functional response curve of subyearling Chinook salmon fit to a range of *Daphnia pulex* densities from laboratory trials and the 95% confidence interval about the mean (region between dashed lines).

Across the entire range of *Daphnia* densities that we evaluated and a subset of ecologically relevant densities, *AIC*_C_ scores provided strong evidence for the non-linear model over the linear model. Our non-linear model across all densities was: *C* = 29.858 *P* * (4.271 + *P*)^-1^, while for the ecologically relevant range it was: *C* = 28.561 *P* * (3.530 + *P*)^-1^. In both cases, a Type-II functional response was supported over the linear Type-I response based on *AIC*_*C*_ scores (Fig 2 and Table 3). Also, both the Type-I and Type-II ecologically relevant density models were superior to their respective Type-I and Type-II model over the entire range of *Daphnia* densities based on lower *AIC*_*C*_ scores. Both non-linear models had asymptotes that were about 30 · L^-1^, which is about the maximum *Daphnia* reported in the literature. In fitting the original linear model, we had one point (Density = 161.1 · L^-1^, Consumption = 48.2 · min^-1^) that exhibited a high degree of influence (Cook’s *D* value = 3.7). Another point (Density = 97.5 · L^-1^, Consumption = 20.5 · min^-1^) had a Cook’s *D* value of 0.523. Although these points had ecologically irrelevant (high) densities compared to empirical densities from the literature (Table 4), we retained these data points because they had no effect on the overall outcome of model selection based on *AIC*_*C*_ score when removed.

**Table 3:** Parameter estimates for linear and nonlinear functional response models describing the relation between *Daphnia* density and subyearling Chinook salmon consumption. The two data sets are from all the data and an ecologically relevant subset (*Daphnia* densities < 40 · L^-1^).

	All *Daphnia* densities		Ecologically relevant *Daphnia* densities	
Data set	Type-I (Linear model)	Type-II (Nonlinear model)	Type-I (Linear model)	Type-II (Nonlinear model)
Slope (SE)	0.422 (0.07)	N/A	1.171 (0.13)	N/A
Corrected df	33	33	28	28
β_o_ (SE)	N/A	29.858 (3.37)	N/A	28.561 (3.63)
β_1_ (SE)	N/A	4.271 (2.14)	N/A	3.530(1.84)
Model *p* value	< 0.001	< 0.001	< 0.001	< 0.001
*n*	34	34	29	29
*k*	2	3	2	3
RSS	7515.1	1799.2	2896.7	1131.2
*R*^2^	0.55	N/A	0.76	N/A
*AIC*_*C*_	187.9	141.7	138.0	113.2

**Table 4:** Maximum July and August *Daphnia* densities from various literature sources, predicted subyearling consumption rates (# · hr^-1^) from a laboratory derived functional response curve in this study, and estimated time to satiation (h) from McNary and John Day reservoirs, lower Columbia River, 1982–2010, based on previously derived evacuation rates from Haskell et al. [14]. Mean *Daphnia* lengths used for 1982 were 1.4 mm in July and 1.5 mm in August. All others used a *Daphnia* length of 1.2 mm.

Collect Year	July Density (# · L^-1^)	Predicted Consumption (# · hr^-1^)^5^	Time to Satiation (h)^6^	August Density (# · L^-1^)	Predicted Consumption (# · hr^-1^)^5^	Time to Satiation (h)^6^
1982^1^	2.6	677.9	28.7	18.5	1455.5	12.9
1994^2^	0.7	252.3	118.8	19.0	1462.7	23.3
1995^2^	0.1	41.0	731.1	2.6	677.9	50.3
1996^2^	0.4	153.4	195.3	2.5	661.5	51.5
1994^3^	1.7	510.1	58.7	19.6	1471.0	23.2
1995^3^	0.4	153.4	195.3	39.5	1616.7	21.1
2009^4^	0.2	80.1	373.9	0.1	41.0	831.4
2010^4^	0.1	41.0	831.4	2.0	571.4	59.6

^1^Rondorf et al. [12]

^2^Haskell et al. [10]

^3^Gilbreath et al. [34]

^4^Emerson et al. [11]

^5^This study

^6^Used evacuation rates from Haskell et al. [14]

### Empirical *Daphnia* densities

Our comparisons of *Daphnia* densities from the literature to consumption rates from our Type—II functional response curve predicted subyearling consumption ranging from 0.29 to 26.95 *Daphnia* · min^-1^. Previous studies of *Daphnia* densities in McNary and John Day reservoirs indicated increasing *Daphnia* densities through June and July, with peaks in early August followed by sharp declines starting in mid-August. Specific *Daphnia* densities ranged from 0.1 to 2.6 · L^-1^ in July and from 0.1 to 39.5 · L^-1^ in August. *Daphnia* densities in July and August, corresponded with *Daphnia* consumption predicted from our functional response curve ranging from 0.29 to 24.37 *Daphnia* · min^-1^. Based on our Type-II functional response, the greatest *Daphnia* densities reported by Gilbreath et al. ([36]; 39.54 · L^-1^), subyearling consumption would support a feeding rate of 26.95 *Daphnia* · min^-1^. In July through September of 1968 and 1969, subyearlings predominately consumed similar amounts of *Daphnia* but at densities that were lower. In 1968, subyearlings exhibited an average of 1 *Daphnia* stomach^-1^ in July, 259 *Daphnia* · stomach^-1^ in August, and 971 *Daphnia* · stomach^-1^ in September. In 1969, subyearlings exhibited an average of 3 *Daphnia* · stomach^-1^ in July, 647 *Daphnia* · stomach^-1^ in August, and 961 *Daphnia* · stomach^-1^ in September. However, during this study, *Daphnia* densities peaked in August at 2.5 · L^-1^ in 1968 and 1.7 · L^-1^ in 1969 [38].

### Subyearling bioenergetics growth simulations

Our bioenergetics runs using empirical data collected by Haskell et al. [14] in July and August, 2013 predicted negative growth under both simulations (Table 5). Increases in growth rate from July to August, although still negative, were associated with decreases in daily ration, about a 1.5°C increase in water temperature, and a change from planktivory to piscivory. In July when subyearlings were feeding on *Daphnia*, subyearling growth ranged from -0.23 to -0.29 g · d^-1^ and when subyearlings switched to juvenile American shad prey, their growth ranged from -0.06 to -0.10 g · d^-1^.

**Table 5:** Results of four-day bioenergetics simulations for subyearling Chinook salmon conducted for subyearling Chinook in John Day Reservoir, Columbia River during July (primarily feeding on *Daphnia*) and August (primarily feeding on juvenile shad) 2013. Initial start weight and daily ration (*D*) values were obtained from Haskell et al. [14].

Date	Water Temp^1^ (°C)	Start Weight^2^ (g)	Daily Ration^2^ (*D*; g)	Specific Growth (g · g · d^-1^)	End Weight (g)	Growth (g · d^-1^)	Growth Efficiency (*GE*; %*)*
Jul 21	20.57	20.10	2.21	-0.013	19.84	-0.257	-11.7
Jul 22	20.59	19.84	2.18	-0.013	19.59	-0.257	-11.8
Jul 23	20.88	19.59	2.15	-0.014	19.31	-0.227	-12.8
Jul 24	21.06	19.31	2.12	-0.015	19.03	-0.285	-13.4
Aug 11	21.97	20.00	1.70	-0.003	19.95	-0.053	-3.1
Aug 12	21.94	19.94	1.69	-0.003	19.90	-0.051	-3.0
Aug 13	22.00	19.88	1.69	-0.003	19.84	-0.056	-3.3
Aug 14	22.16	19.81	1.68	-0.004	19.77	-0.071	-4.2

^1^DART[42]

^2^Haskell et al. [14]

**Fig 3:**
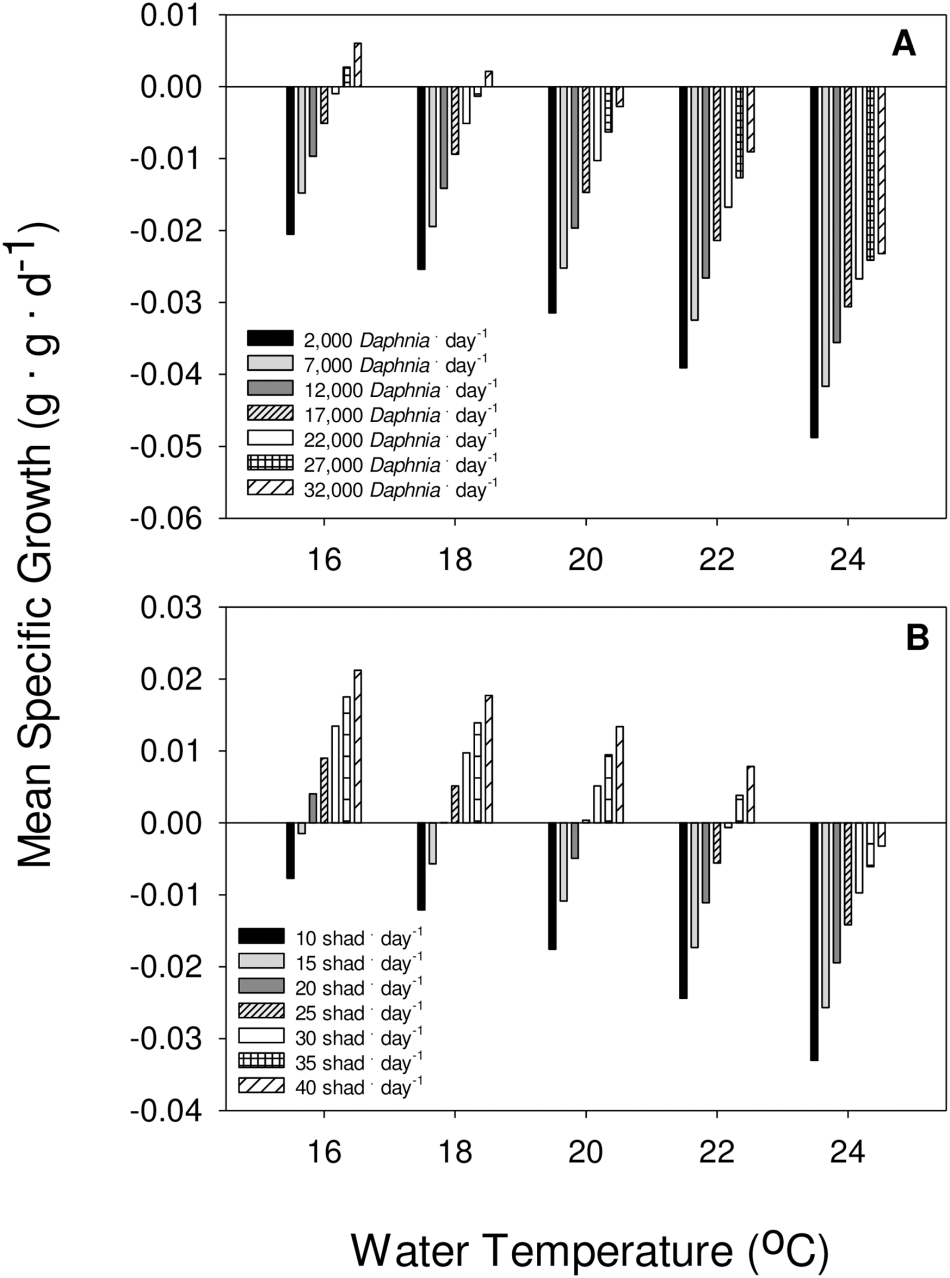
Bioenergetics modeled specific growth (g · g · d^-1^) of subyearling Chinook salmon at varying consumption rates ranging from 2,000 to 32,000 *Daphnia* · day^-1^ (A) and juvenile American shad ranging from 10 to 40 shad. day^-1^ (B) at water temperatures ranging from 16 to 24°C.

Growth potential for subyearlings varied considerably across the range of temperatures, prey densities, and prey types that could be experienced during the summer in these reservoirs. Subyearlings did not exhibit positive growth at water temperatures above 22°C, irrespective of prey type or amount (Fig 3). When feeding on *Daphnia*, subyearlings needed to consume about 27,000 *Daphnia* · day^-1^ (representing 90% *C*_*max*_) to achieve positive growth at 16°C, but could not achieve positive growth when feeding on *Daphnia* at 20°C. When feeding on juvenile shad, subyearlings needed to consume 20 shad · day^-1^ to exhibit positive growth at 16°C (Fig 3). At 20°C, subyearlings needed to consume about 25 shad · day^-1^ (representing 60% *C*_*max*_) to achieve positive growth. At 24°C, subyearlings could not consume enough shad to achieve positive growth (Fig 3).

Our stomach capacity and time to satiation data indicate that during July, subyearlings would need to feed considerably longer to achieve satiation because *Daphnia* densities were lower than in August. We estimated a maximum stomach capacity of 1.54 g for a 20 g subyearling. With this stomach capacity, subyearlings could consume a maximum of 17,380 *Daphnia* (1.2 mm). Using the range of empirical *Daphnia* densities from the literature, subyearlings feeding in July could achieve satiation in 28.7 to 831.4 h (Table 4). Based on August *Daphnia* densities as reported in the literature, subyearlings could achieve satiation in as little as 12.9 h at densities approaching 20 · L^-1^. However, a nearly two-fold increase of *Daphnia* density from 20.0 to 39.5 · L^-1^ was not associated with a reduction in satiation time.

## Discussion

Our feeding trials indicated that subyearling consumption of *Daphnia* increased with prey density and exhibited a Type-II response. Although there are no functional response models for juvenile Chinook salmon, other juvenile salmon models have been developed using laboratory feeding trials. Koski and Johnson [29] estimated the functional response of kokanee salmon (*O*. *nerka*) fingerlings to *Daphnia* density under varying light conditions and compared them to natural conditions in Blue Mesa Reservoir, Colorado. While kokanee had a linear response at low light levels typical of crepuscular periods, they exhibited a Type-II response at higher light levels. Koski and Johnson [29] reported a linear response for kokanee feeding at 0.1 lx but a Type-II response at 15 lx and 30 lx- light levels that occur during crepuscular periods when feeding is highest in Blue Mesa Reservoir. In our trials, light was held constant but varied slightly across tanks from 11.8–24.8 lx. Given the similarity of our light levels to Koski and Johnson [29], we surmise that light differences had little effect on subyearling feeding rate in our trials. In the wild, subyearling consumption of *Daphnia* is highest during late afternoon [14]. Subyearlings can probably forage on *Daphnia* at a higher rate than on copepods given the less evasive nature of *Daphnia*. Juvenile chum (*O*. *keta*) and pink salmon (*O*. *gorbuscha*) had far lower consumption rates feeding on copepods [30].

Subyearlings in lower Columbia River reservoirs feed at a high rate but are unlikely to reach satiation in July because of the relatively small size of *Daphnia* in the lower CRB. With consumption of 1.2 mm-sized *Daphnia*, a 20 g subyearling could reach satiation by consuming 17,380 *Daphnia*. Using an empirical consumption estimate (which accounts for evacuation rate) of 11.0% body weight, subyearlings feeding on *Daphnia* in July could reach satiation in 16.7 h, an unlikely scenario given that subyearlings feed little at night [14]. For comparison, a 20 g kokanee in Blue Mesa Reservoir could reach its stomach capacity by consuming 9,386 *Daphnia* owing to the larger size of the *Daphnia* there (1.84 mm; [44]). Although size selective planktivory can account for smaller-sized zooplankton [46], rivers also favor small bodied zooplankton taxa which have shorter generation times [47,48].

Empirical data collected in John Day Reservoir in 2013 indicated that when feeding almost exclusively on *D*. *retrocurva* (97% of total prey) during the time of maximum consumption (1700 hr), 20.1 g subyearlings consume 11% of their body weight per day [14]. During peak *Daphnia* density in August of about 20 to 40 · L^-1^, a 20.0 g subyearling Chinook salmon would need to forage from 12.9 to 23.3 h to reach satiation when consuming *Daphnia*. Time to satiation was 12.9 h at a *Daphnia* density of 18.5 · L^-1^, but 23.3 h at a *Daphnia* density of 19.0 · L^-1^. This difference in satiation time despite only a small *Daphnia* density difference was due to larger *Daphnia* in 1982 (1.5 mm) relative to 1994 (1.2 mm). When *Daphnia* densities were highest (39.5 · L^-1^), as observed by Gilbreath et al. [34], time to satiation was still 21.1 h due to subyearlings feeding beyond the asymptote on the flat part of the Type-II functional response curve. Due to the relatively high *Daphnia* densities observed in some studies during August, subyearlings can feed at a high level that approaches satiation, however, when *Daphnia* densities are low as in July, subyearlings cannot reach satiation.

Juvenile American shad are potential competitors of subyearlings, because their presence overlaps in main-stem reservoirs and they consume *Daphnia* [16]. Our functional response curve indicates that at *Daphnia* densities greater than 30 · L^-1^, reductions in *Daphnia* density have little effect on subyearling consumption. It is not until *Daphnia* density decreases below 20 · L^-1^ that subyearlings exhibit reduced consumption. Our data indicate that prey-limiting (*Daphnia*) conditions are more likely in July, when *Daphnia* densities are low, than in August. During July, most young shad have yet to develop beyond the larval stage [49]. However by August, subyearlings have switched from feeding on *Daphnia* to juvenile shad. We conclude that while *Daphnia* are an important prey item of both subyearlings and shad, they are utilized only briefly by juvenile shad in late July and early August when shad are about 20–40 mm. Juvenile shad mostly consume copepods in August and September [10].

We used functional response to predict subyearling consumption using changes in *Daphnia* density. Given the temporally and spatially dynamic nature of predator-prey interactions in lower Columbia River reservoirs, additional functional curves for other important predators and prey would be useful. For example, subyearlings prefer *D*. *retrocurva* over the invasive calanoid copepod, *P*. *forebsi* during late summer [50]. However, despite the numerical dominance of *P*. *forebsi*, subyearlings switch to piscivory on juvenile shad instead of consuming *P*. *forebsi* after the mid-summer decline of *Daphnia* [14]. Subyearlings may find it more energetically beneficial to switch from *Daphnia* to juvenile American shad rather than *P*. *forebsi*. Juvenile shad reduce *Daphnia* production otherwise available to subyearlings and may be able to exploit *Daphnia* at higher rate than juvenile salmon. A comparative functional response approach could be used to understand if nonnative juvenile shad consume *Daphnia* at a higher rate than juvenile salmon- a potential measure of competition [51–53], or whether subyearlings consume native *Daphnia* at a higher rate than nonnative copepods (e.g., *P*. *forebsi*) as suggested by others [50,54]. Additional functional response curves could be developed for the period after *Daphnia* decline and subyearlings begin feeding on juvenile shad and shad themselves begin feeding on copepods [14].

Our bioenergetics modeling predicted that subyearlings would need to consume as many as 32,000 *Daphnia* to grow. Similarly large predictions for other salmonids are not unprecedented. Kokanee need to consume 6 *D*. *pulex* per second (21,600 · h^-1^) to reach observed growth in Blue Mesa Reservoir [44]. Examination of subyearling stomach contents from the CRB indicated individual stomachs containing as many as 2,878 *Daphnia* (N = 16, median = 1,180) [14]. Lastly, we observed consumption rates of nearly 50 · min^-1^ in our functional response trials. At this rate, subyearlings could consume 32,000 *Daphnia* in about 11 h. We believe that the upper range of *Daphnia* consumption we modeled is feasible despite the resulting growth constraints.

Using bioenergetics, we predicted subyearling growth across a range of prey and temperature scenarios that subyearlings could experience while migrating seaward through the lower Columbia River. Our simulations broadly indicated that while the energy density of juvenile American shad prey is greater than *Daphnia*, subyearlings would need to consume about 35 shad · day^-1^ to exhibit positive growth at 22°C or higher. Although we did not conduct functional response experiments using juvenile shad prey which would have allowed us estimate maximum consumption rates as we did with *Daphnia*, others have reported subyearlings consuming up to 20 larval and juvenile Pacific sand lances (*Ammodytes hexapterus*) per day [55]. While subyearlings derive an energetic benefit with a shift from planktivory to piscivory in late summer, their growth is limited by the constraints of migrating under high water temperatures. The trends we demonstrated are consistent with decreasing optimal temperatures and thermal tolerances as daily rations decline [25]. However, because increased *Daphnia* densities are associated with higher water temperatures, subyearlings face a trade-off between increased foraging opportunity and their thermal tolerance [56].

The data supporting our bioenergetics model runs [14] have some uncertainties. Uncertainty surrounding bioenergetics parameters, especially for salmonids, has been conducted using both individual parameter perturbation and Monte Carlo simulations [57] indicating that the mass-dependent parameters for *C*_*max*_ (CA or CB) or respiration (RA or RB) are typically the most sensitive. The Chinook salmon bioenergetics model has been corroborated with predictions of consumption or growth typically within 5–10% of measured responses in lab studies [58] and when compared to independent field estimates [59]. These studies indicate that most of the uncertainty results from population-specific inputs to the model rather than the physiological parameters. For our model runs, these inputs were daily ration (consumption), prey and predator energy densities, and thermal experience. We used energy densities from the literature and modeled growth using empirical water temperatures and also using a broad range of temperatures subyearlings could experience. We addressed the primary source of uncertainty by developing a functional response curve with a 95% confidence interval.

Our second set of bioenergetics simulations had subyearlings consuming 100% *Daphnia* and 100% juvenile shad. This *Daphnia* percentage was supported by empirical data from the forebay of John Day Reservoir where subyearlings consumed about 98% *Daphnia* [14]. Although we believe that subyearlings predominately consume *Daphnia* as supported in the literature from reservoir habitats, it is possible that their diet is more variable in upstream portions of the reservoir and over longer time frames than the 3-day period that was sampled. The empirical data also indicated that subyearlings consume close to 100% juvenile shad by weight (71% by number; [14]). These data were similarly collected over a 3-day period in the John Day Reservoir forebay during August 2013. Although we used simplified diet scenarios in our simulations, we believe that they were representative of the two important prey items of later migrating subyearlings.

Subyearlings move though John Day Reservoir fairly quickly but compromised growth opportunity there could have lasting effects. The mean travel times (2006–2015) of subyearlings through John Day Reservoir ranged from 3.1 to 5.3 days [60] with only small differences between wild (Mean = 4.6, SE = 0.2) and hatchery origin (Mean = 4.2, SE = 0.2) subyearlings. Over the 10 years, subyearlings exhibited a mean travel time of 3.1 days (SE = 0.02) in July and 4.0 d (SE = 0.15) in August. Based on these travel times along with the consumption and growth data presented here, we infer that the “average” subyearling could lose 3.4% of its body weight in July and 1.2% of its body weight in August while migrating through John Day Reservoir. Although not widely demonstrated in field studies, young salmon can temporarily withstand periods of negative growth resulting from unfavorable temperatures [61]. However, the long term consequences of these periods on survival and smolt-to-adult- ratios (SAR’s) are unknown.

Although the topic of how much energy loss equates to mortality or debilitation of fish has not yet received adequate attention in the literature, a suggestive convergence in relative energy loss-mortality has begun to emerge for juvenile and adult salmonids. Mortality has been associated with a decline to about 40–45% of initial energy content [25,62]. When compared to the 1.2–3.4% loss in body mass, which is analogous to energy loss, juvenile Chinook migrating through lower CRB reservoirs are not in immediate danger of starvation. However, compromised growth could have serious consequences for the survival of early marine life stages due to strong size-selective mortality reported for yearling and subyearling Chinook [3,4,63,64].

Human mediated habitat alterations have delayed the seasonal timing of subyearling migration through John Day Reservoir. Whereas historically, subyearlings migrated through the lower Columbia River in June and July [65], most now migrate starting in July, with Snake River subyearlings migrating seaward into August. Prior to 2005, later-migrating ESA-listed Snake River subyearlings were nearly all collected and transported downstream, leaving fewer fish in the river. Changes brought forth in the Biological Opinion for operation of the Federal Columbia River Power System created more spill to aid juvenile migrants during July and August [66]. Despite shad-mediated *Daphnia* reductions, subyearlings are feeding at a relatively high rate in the lower Columbia River during July and August [14]. By switching from lower energy density *Daphnia* to higher energy density fish prey, subyearlings can partially compensate for the metabolic costs of higher water temperature. However, empirical data suggests that they are not growing despite this switch and our modeling indicates that subyearlings need to consume 35 shad · day^-1^ to achieve positive growth at 22°C.

Water temperatures of 22°C during July and August are prevalent not only in John Day Reservoir, but throughout the lower Columbia River [39]. One management strategy for fish during this time is increasing spill to aid downstream migration. Our data suggest that water temperatures above 20°C coupled with reservoir prey taxa of lower energetic quality provide little growth opportunity for subyearlings during this time. While our results indicate compromised growth in reservoir habitats, the long-term repercussions to salmon populations in the Columbia River Basin are unknown. The topic of how much energy loss equates to mortality or debilitation of fish has not yet received adequate attention.

## Supporting information

S1 Table Results of functional response trials for subyearling Chinook salmon and *Daphnia*. (PDF)
