# Supplementary material for: Linking functional response and bioenergetics to estimate juvenile salmon growth in a reservoir food web
Source: PLoS One. 2017 Oct 11;12(10):e0185933. doi: 10.1371/journal.pone.0185933 (PMC5636121; doi:10.1371/journal.pone.0185933)
Supplement: S1 Table — (PDF) [file pone.0185933.s001.pdf]

Appendix Table A.1. –Results of functional response trials for subyearling Chinook salmon and *Daphnia*.

Trial Date	Trial Temp (^{o}C)	Fish Length (mm)	Fish Weight (g)	Total *Daphnia* leftover	Prey Density (# · L^{-1})	Total *Daphnia* consumed	Consumption Rate (*Daphnia* · min^{-1})
10/20	20.5	115	15.1	856	15.4	371	37.1
10/20	20.5	120	19.2	1,049	18.8	251	25.1
10/20	20.5	130	19.4	662	11.9	262	26.2
10/20	20.5	128	21.1	988	17.7	226	22.6
10/20	20.5	119	17.2	667	11.4	246	24.6
10/20	20.5	123	20.3	411	7.7	178	17.8
10/27	20.4	129	20.7	5,431	97.5	205	20.5
10/27	20.4	139	25.5	340	6.1	121	12.1
10/27	20.4	139	27.8	477	8.6	296	29.6
10/27	20.4	128	21.8	8,992	161.4	482	48.2
10/27	20.4	127	22.5	206	2.4	119	11.9
10/27	20.4	130	22.3	116	2.8	180	18.0
10/27	20.4	128	21.8	334	5.5	138	13.8
11/10	20.3	137	24.6	12	0.1	7	0.7
11/10	20.3	130	22.8	40	0.3	35	3.5
11/10	20.3	124	21.5	22	0.5	17	1.7
11/10	20.3	127	20.8	33	0.7	80	8.0
11/10	20.3	125	19.9	76	0.9	127	12.7
11/10	20.3	132	24.1	172	2.0	58	5.8
11/10	20.3	132	23.4	52	1.1	55	5.5
11/21	20.4	135	26.8	525	9.4	85	15.0
11/21	20.4	125	20.5	1,291	23.2	406	40.6
11/21	20.4	143	30.1	784	14.1	164	16.4
11/21	20.4	129	23.1	847	15.2	187	18.7
11/21	20.4	130	23.7	855	14.6	118	11.8
11/21	20.4	130	24.3	981	18.5	179	17.9
11/21	20.4	128	20.3	1,310	23.0	235	23.5
11/21	20.4	139	29.2	664	10.9	210	19.4
1/12	20.3	125	21.9	1,693	30.4	311	31.1
1/12	20.3	145	36.0	2,793	50.1	173	17.3
1/12	20.3	142	29.8	2,761	48.4	189	18.9
1/27	20.3	141	33.3	3,429	64.6	354	35.4
1/27	20.3	142	32.9	2,258	38.7	159	15.9
1/27	20.3	143	33.6	1,303	23.4	276	27.6
